# Prevalence and factors associated with burnout among nurses in Jeddah: a single-institution cross-sectional study

**DOI:** 10.1186/s12912-022-01070-2

**Published:** 2022-10-26

**Authors:** Jumanah T. Qedair, Renad Balubaid, Raghad Almadani, Suzana Ezzi, Tarteel Qumosani, Rania Zahid, Turki Alfayea

**Affiliations:** 1 College of Medicine, King Saud Bin Abdulaziz University for Health Sciences, Jeddah, Saudi Arabia; 2 King Abdullah International Medical Research Center, Jeddah, Saudi Arabia; 3 Medical Oncology Department, King Abdullah Specialist Children Hospital, Riyadh, Saudi Arabia; 4 College of Medicine, King Saud Bin Abdulaziz University for Health Sciences, Riyadh, Saudi Arabia

**Keywords:** Burnout, Nurses, Saudi Arabia, Stress, Burnout prevalence

## Abstract

**Background:**

Health care workers, especially nurses, experience significantly elevated levels of emotional and social stressors in the work environment. Therefore, nurses develop high levels of burnout as the stress persists for prolonged periods. The main purpose of this paper is to measure burnout levels amongst nurses and find a relation between burnout levels and demographic factors.

**Methods:**

This descriptive cross-sectional study was held from the 23rd of May till the 6th of September 2021 in King Abdulaziz Medical City of National Guard Health Affairs (KAMC-JD) in Jeddah. Data had been collected voluntarily from the nurses through electronic surveys that included demographic data and the Maslach Burnout Inventory (MBI) that measures three dimensions of burnout which are emotional exhaustion (EE), depersonalization (DP), and personal accomplishment (PA). The association between demographic variables and burnout had been studied using the Fisher Exact test and binary logistic regression.

**Results:**

Out of the 1300 nurses working in KAMC-JD, 250 completed the survey. Burnout had been detected in 112 nurses (44.8%). Out of the 250 nurses, 26.4% were found to be at a high risk of burnout, which means they have high or moderate scores of EE and DP, with low ones in PA. The majority of the participants had high burnout levels in each of burnout components, and 99.6% of them scored high in at least one of the three dimensions. Level of burnout was significantly correlated to certain demographic factors which were the nationality (*p-*value = 0.01) and working unit (*p-*value = 0.02). On the other hand, there was no significant association between burnout and age, gender, or marital status.

**Conclusion:**

This study proves that a high percentage of nurses fell victims to burnout with a strong association between certain demographic data namely nationality as well as working unit and burnout levels. Taking into consideration the negative impact of burnout on both nurses and patients, conducting more studies about burnout among nurses, its effect on them, and the risk factors behind it is needed. Early treatment and management are also recommended to avoid the undesirable outcomes.

## Introduction

Burnout describes an occupational psychosocial syndrome that results from the poor management of elevated levels of emotional and social stress in the workplace for prolonged time periods [1]. As per the World Health Organization (WHO) definition, it consists of three main dimensions or observed signs: exhaustion, cynicism, and inefficacy [2]. Thus, those who suffer from burnout typically feel emotionally drained, develop negative attitudes towards their colleagues, and feel incompetent or fail to accomplish their tasks and responsibilities effectively, respectively [3].

Although burnout was previously defined as a single entity, recent studies have classified burnout into three types to distinguish different symptoms and adjust more effective methods of therapy for each. While “frenetic” burnout is common in extremely ambitious individuals who constantly challenge themselves to the point of burnout, the “under-challenged” type is observed in persons whose jobs are dull and repetitive, leading them to feel unaccomplished and indifferent. Finally, individuals who easily give up and lose control at times of stress are those who exhibit the “worn-out” type of burnout [1].

The healthcare field, and in particular the nursing environment, could be a highly stressful workplace due to its demanding responsibilities, almost diminished control over work nature, lack of social support, and long working shifts [4–6]. Additionally, there are other stressors facing nurses in their working settings, such as dealing with pain, patient deaths, and breaking bad news to patients [7]. Nurses can also get exposed to emotionally demanding circumstances in their personal life. This work-life interference can lead to emotional exhaustion, which in return result in burnout [8]. As a meta-analysis of a global sample of 45,539 nurses of different specialties highlighted, the prevalence rate of burnout symptoms is about 11.3% among nurses [9].

In the Middle East, nurses reported the highest level of burnout among healthcare providers [10]. Rates of burnout among nurses of primary care centers of Saudi Arabia reached up to 89% of nurses exhibiting at least one subscale of burnout [11], where the most common work-related stressors were high workload and shortage of staff [6, 7]. Such factors can result in emotional exhaustion and burnout in nurses, which have proven adverse effects on the quality of care provided, patient safety, and job satisfaction [5, 6]. Moreover, Burnout has not only adverse influence on individual providers and patient care, but it can also adversely affect the entire healthcare system [12].

In Saudi Arabia, the Ministry of Health provides the citizens with medical services at all levels, works on preventing diseases, improves the overall public health, establishes regulations and laws that rule the public and private health sectors with a continuous assessment of the outcomes and performance, in addition to fostering interest in research field, academic training, and aspects of health investment [13]. In the capital city, Riyadh, the ministry offers 60% of healthcare services, while nongovernmental sectors deliver 23% and other governmental facilities offer 17%. The Saudi Arabian healthcare system is currently undergoing changes and development. This resulted from the Ministry of Health's new vision and the creation of a national health strategy to address and overcome the issues.

The Saudi Arabian health care system requires an urgent development and launching of new projects for enhancement with a particular emphasis on the basic healthcare reforms. These reforms necessitate the demanding situation in numerous aspects of health and overall health to be addressed including financing, scope, structure, infrastructure, increased demand, increased expenses, personnel, inequitable access to the services, safety and quality of provided services, overwhelming burden of chronic diseases, referral system, information systems, and the leadership and supervision concerns [14]. In Jeddah, in which this study was held, the total number of nurses working in Ministry of Health is 7344, 79% of which are females [15]. Working conditions mainly depend on the location, number of staff, beds, and interpersonal skills.

According to the Saudi statistical yearbook in 2020, the rate of nurses per 10,000 of the population was 4.1. additionally, the number of beds could be a great indicator of the workload and the amount of stress nurses are facing. In Jeddah, 8,785 hospital beds are in service while in Riyadh 20,802 are available [15]. Considering that both cities are highly populated with increasing demands on hospital beds and healthcare services, the difference is huge between the two cities. Also, worthy of mentioning that there is a good number of benefits for healthcare workers, especially, in National Guard Health affairs, which KAMC is part of, such as annual, and Eid/holiday leaves, in addition to only nursing-related benefits including 10 day paid leave and airline ticket [16].

There is a shortage in literature about the prevalence of burnout among nurses working in Saudi Arabia and the factors associated with it. Therefore, the purpose of this research was to measure the levels of burnout amongst nurses working in King Abdulaziz Medical City in Jeddah (KAMC-JD) and to explore the associated predictors.

## Methods

The questionnaire of this cross-sectional study was composed of two sections. The first section targeted the demographic data of the participants. The second section was the Maslach Burnout Inventory- Human Services Survey for Medical Personnel (MBI-HSS (MP)), which is highly validated as a measure for burnout among healthcare workers including nurses, as defined by the World Health Organization (WHO). It comprises 22 symptom items according to the following dimensions: emotional exhaustion (EE), depersonalization (DP), and personal accomplishment (PA). The 9-item Emotional Exhaustion (EE) sub-scale measures the effect of the healthcare worker’s occupation on their emotions. The 5-item Depersonalization (DP) sub-scale assesses the impersonal response towards recipients of the healthcare worker’s service and care. The final 8-item Personal Accomplishment (PA) sub-scale examines the feelings of success and achievement in the healthcare worker’s occupation. The scoring system of all the MBI’s items was a 7-level frequency rating that ranges from “never” to “every day”. Higher scores in EE and DP subscales and lower scores in PA subscale indicate higher levels of experienced burnout. In addition, to be able to identify burnout presence and absence, burnout scores were categorized into three categories of low, intermediate and high scores, that are shown in (Table 1) and then based on that into four groups: a group suffering from burnout, a group at high risk, and another neither suffering nor at high risk currently.

**Table 1:** Classification of burnout subscales

Level of Burnout	Emotional Exhaustion	Depersonalization	Low Personal Accomplishment
Low	$\le 16$	$\le 6$	$\le 31$
Moderate	17–26	7–12	32–38
High	$\ge 27$	$\ge 13$	$\ge 39$

The sample size was calculated by using the Raosoft software www.raosfoft.com/samplesize.html. The total number of the nurse at KAMC-JD for 2021 is 1300 nurses. The required sample size was estimated at a 95% confidence level with a margin of error of ± 5%. The required minimum sample size was estimated to be 297. However, any response rate higher than 10% was considered reasonably acceptable among such healthcare workers with busy schedule. A purposive sampling technique was followed to recruit nursing professionals.

The authors contacted the nurse manager to request electronically distributing the questionnaire to nurses from all KAMC’s departments (surgical and medical wards) including Oncology Center, Cardiology Center, Bone Marrow Plantation Center, Emergency, Intensive Care Unit, Primary Healthcare Clinic, and Burn Unit. After approving the request, the nurse manager was formally asked to send online invitation emails containing an explanatory statement, the consent form, and the self-administered Google survey to all the aforementioned nurses. The data were collected over a 3-month period, starting from the 23rd of May until the 6th of September 2021. The authors regularly sent reminders to the manager to enhance the response rate when it was noticed to be declining.

### Ethical consideration

The Institutional Review Board (IRB) approval was obtained from King Abdullah International Medical Research Center (KAIMRC), and all responses were kept fully confidential with authorized access only. All participants gave their consent to be involved in this study, and they were informed that no identifier information was to be asked.

### Statistical analysis

Descriptive statistics have been presented using counts, proportions (%), median (min–max), mean and standard deviation whenever appropriate. The association between burnout level and the socio-demographic characteristics of the nurses were studied using Fisher's exact test followed by binary logistic regression. *P* < 0.05 was considered statistically significant. Normality, statistical interactions, and collinearity (i.e., variance inflation factor) were also assessed with the Kolmogorov–Smirnov and Shapiro Wilk test. As the data follow the non-normal distribution, non-parametric tests were applied. Correlation procedures were performed to determine the linear relationship between EE, DP, and PA scores. MBI tool was found to be reliable in our research with an Alpha Cronbach of 0.85. All data analyses were performed using Statistical Packages for Software Sciences (SPSS) version 26 Armonk, New York, IBM Corporation.

## Results

### Demographic profile

Of the 1300 eligible nurses who were invited to fill the survey, 250 participated in the study (*n =* 250), corresponding to a response rate of 19.23%. Inpatient department nurses gave the maximum response rate reaching up to 79.6% of the responses. Nationality-wise, non-Saudi nurses gave the highest number of responses forming 76.0% of the sample. The majority of the participants, 233 (93.2%), were females, and 54.1% of them were married. Sample had been divided into 2 age groups and the majority, 64.4%, were between 18 and 38 years old (Table 2).

**Table 2:** Demographic characteristics of the nurses and their association with burnout level

Study Data	Overall N (%) (*n =* 250)	Burnout			*P-*value
		**Yes (*n =* 112)**	**High risk (*n =* 69)**	**No (*n =* 69)**	
		**N (%)**	**N (%)**	**N (%)**	
Age group					
• 18–38 years	161 (64.4)	76 (47.2)	40 (24.8)	45 (28.0)	0.40
• > 38 years	89 (35.6)	36 (40.4)	29 (32.6)	24 (27.0)	
Gender					
• Male	17 (06.8)	9 (52.9)	5 (29.4)	3 (17.6)	0.73
• Female	233 (93.2)	103 (44.2)	64 (27.5)	66 (28.3)	
Nationality					
• Saudi	60 (24.0)	37 (61.7)	14 (23.3)	9 (15.0)	0.01*
• Non-Saudi	190 (76.0)	75 (39.5)	55 (28.9)	60 (31.6)	
Marital status					
• Married	131 (52.4)	59 (45.0)	42 (32.1)	30 (22.9)	0.13
• Unmarried	119 (47.6)	53 (44.5)	27 (22.7)	39 (32.8)	
Working unit					
• Inpatient service	198 (79.2)	97 (49.0)	48 (24.2)	53 (26.8)	0.02*
• Outpatient service	52 (20.8)	15 (28.8)	21 (40.4)	16 (30.8)	

^§^*P*-value has been calculated using Fisher's exact test

^*^ Significant at *p* < 0.05 level

## The MBI tool and the correlation between the 3 subscales

**Fig. 1:**
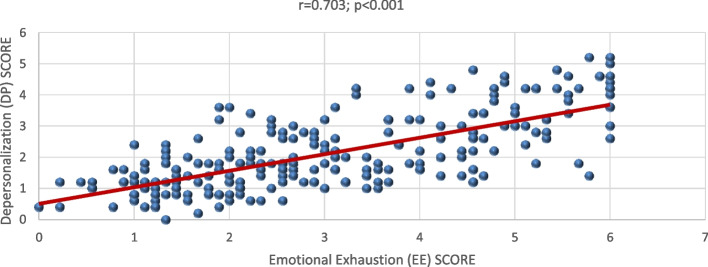
Correlation between Emotional Exhaustion (EE) and Depersonalization (DP)

**Fig. 2:**
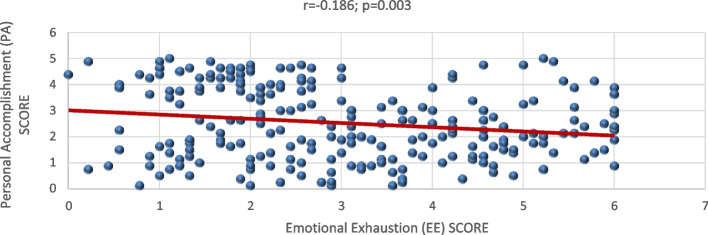
Correlation between Emotional Exhaustion (EE) and Personal Accomplishment (PA**)**

**Fig. 3:**
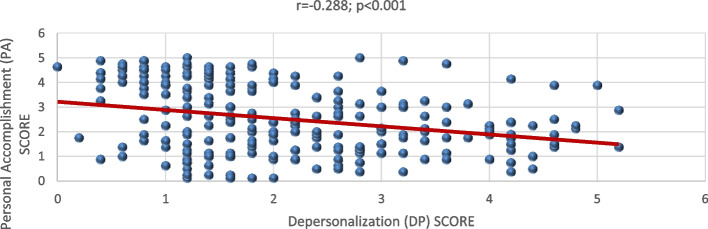
Correlation between Depersonalization (DP) and Personal Accomplishment (PA)

The value of Cronbach’s Alpha for the used instrument equaled 0.85 showing that it was reliable. While for the correlation between the subscales, there had been a significant positive relation between each of them with a *p-*value of < 0.001 (*r =* 0.703) between EE and DP, 0.003 *p-* value (*r =* -0.186) between EE and PA, and < 0.001 *p-*value (*r =* -0.288) between DP and PA. These linear relationships among them are shown on Figs. 1, 2, and 3.

## Burnout levels among the nurses

According to the selected definition of burnout that is described as high levels of both EE and DP with low level of PA, it has been found that 44.8% of the subjects are suffering from burnout. In addition, 26.4% were found to be at a high risk of burnout, which means they have high or moderate scores of EE and DP, with low ones in PA. The median scores of MBI’s three subscales (EE, DP, and PA) among the 250 participants were 2.89, 1.80, and 3.62 respectively. In addition, the majority of the scores were high in each of burnout items with PA being the most prevalent (Table 3). Furthermore, 99.4% of the nurses scored high in at least one of burnout subscales.

**Table 3:** Descriptive statistic of MBI subscales

MRI Subscale	Median	Low level n (%)	Medium n (%)	High level n (%)	95% CL	
					**Lower**	**Upper**
**Emotional Exhaustion (EE)**	2.89	44 (17.6)	64 (25.6)	142 (56.8)	2.90	3.29
**Depersonalization (DP)**	1.80	13 (5.2)	58 (23.2)	179 (71.6)	2.00	2.29
**Personal Accomplishment (PA)**	3.62	2 (0.8)	21 (8.4)	227 (90.8)	3.33	3.67

## Association between burnout level and the demographic / occupational characteristic

A significant difference in the level of burnout among the two nationality groups had been detected with the Saudis being more affected by burnout (*p =* 0.01). Analysis also revealed an association between burnout and the working unit showing a higher frequency of burnout among the nurses providing inpatient services (*p =* 0.02). On the other hand, there was no significant association between burnout and age, gender, or marital status (Table 2).

Binary logistic regression had been conducted to all the variables that showed an association with burnout level. Analysis showed that non-Saudi as well as outpatient nurses are associated with a decrease in burnout level whenever compared to Saudis and inpatient nurses with (O*R =* 0.39, 95% CI: 0.21–0.72, and *P* < 0.01) to non-Saudi participants, and (O*R =* 0.40, 95% CI: 0.20–0.79, *p =* 0.01) to outpatient participants (Table 4).

**Table 4:** Predictors of burnout among nurses using binary logistic regression

Predictor	*P-*value	Odds ratio	95% CI	
			**Lower**	**Upper**
**Nationality (Non-Saudi)**	< 0.01*	0.39	0.21	0.72
**Working unit (outpatient)**	0.01*	0.40	0.20	0.79

^*^ Significant at *p* < 0.05 level

## Discussion

On a global scale, nursing profession is known for its overwhelming lifestyle, considering the continuously increased workload and demanding responsibilities. Providing continues personalized patients clinical and emotional care as well as counseling and educating patients and their families in different aspects are examples of assigned daily tasks nurses have to perform. The aforementioned tasks can highly increase the psychological and physical burden on nurses. As a result, unfortunately, the nursing profession is often characterized by frequent job turnover and early burnout as a direct consequence of the stressful nursing environment. According to a recent study that included 50,000 US registered nurses, burnout caused 31.5% of the nurses to quit their job in 2017 [17]. Lu et al., study has shown that in addition to the high occupational stress, nurses had low job satisfaction [18]. This can influence not only the stability of the nurses’ mental status but also the quality of the provided healthcare services to patients. The findings from our study are consistent with these findings.

The prevalence of burnout and its associated factors among nurses working in Saudi Arabia has received little attention in the literature. To assess the prevalence of burnout and associated factors among nurses in all healthcare specialties, this study was performed, which is the first of its kind in Saudi Arabia. This study aimed to assess the level of burnout among nurses working at King Abdulaziz Medical City in Jeddah (KAMC-JD), Saudi Arabia and to investigate the factors associated with burnout among these nurses.

The definition of burnout we used in our study was as stated by Maslach Burnout Inventory- Human Services Survey for Medical Personnel (MBI-HSS (MP)). MBI is a highly validated survey for measuring burnout among healthcare workers including nurses, as defined by the World Health Organization (WHO). Accordingly, burnout has three main dimensions: emotional exhaustion (EE), depersonalization (DP), and personal accomplishment (PA). In our study, these three dimensions were found to be significantly correlated. These correlations indicated that each of EE and DP scores increased by the increased score of the other, while PA score was found to be decreased by the increase of DP and EE scores. Hence, we found that 44.8% of our nurses who scored high in DP and EE had low scores in PA, indicating suffering from some form of burnout.

Many nurses working in Saudi Arabia were detected to have high levels of burnout. A study conducted by Adeeb Shahin et al., demonstrated that about 39% of the nurses had high EE, and 38% had high DP [11]. Another local study including thirty-nine perioperative nurses showed high EE levels in 87.2% of them [19]. Similarly, the nurses in our study scored high in each of the three burnout dimensions, resulting in more than 50% of the nurses with 56.8% and 71.6% prevalence of EE and DP, respectively with DP being the most prevalent. Another national study by Al-Turki et al., elaborated that 45% of the nurses had elevated EE, and 42% of the nurses reported high levels of DP [20].

Burnout was found to be associated with nationality. Al-Turki et al., found that non-Saudi nurses suffered from EE more than Saudi nurses in the study conducted at King Fahad University Hospital (KFUH) [20]. However; in our study, 61.7% of Saudi nurses were more prone to EE and DP compared to other nationalities working in King Abdulaziz Medical City (KAMC-JD). This disparity might be attributable to the difference of bed capacity between KFUH and KAMC-JD, where over 751 beds in KAMC compared to 440 beds in KFUH, also the difference in the size of treatment-target population, which may result in significant variances among nurses working in these two national institutes. A study done by Chen et al., demonstrated that increased average daily patient-nurse ratio was found to be predictable of higher levels of burnout and job dissatisfaction leading some nurses to leave their job [21].

A study conducted by Can ~adas-De la Fuente et al., showed a significant association between experiencing DP and the type of the department the nurse worked in (*p =* 0.005) [22]. Our findings supported this finding as we found that 49% of inpatient units’ nurses suffered from burnout and were more likely to suffer from EE, particularly, compared to their counterparts in outpatient units as a consequence of the challenging work environment and inadequate staffing there. Our findings are also consistent with a local study done in the Western region of Saudi Arabia by Zaki et al., which clearly reported that 71.6% of the Saudi nurses had high levels of burnout making them emotionally drained [23]. A recent meta-analysis supported this and found a significant association between burnout and depression among nurses with positive correlation for the EE subscale of the MBI tool [24].

Thus, to improve nurses’ well-being, which will positively reflect on the quality of healthcare services provided by nurses, decreasing the levels of burnout they experience is crucial, although the actual implications of nurses’ burnout have not been precisely measured in the literature. Our study showed that burnout has been prevalent among nurses working in Saudi Arabia and its associated factors might predict its level. The authors believe that this study will carry a great influence on healthcare policies in the kingdom of Saudi Arabia regarding putting more efforts into enhancing the well-being of nurses working in hospitals with heavy workload. This study can be a starting point to explore burnout among those who have already experienced it and even to sit preventative measurements in the first place through utilizing the studied predictors.

### Recommendations & limitations

Over the last decade, there has been an increase in the awareness and interest in research about the occupational phenomenon of burnout. Moreover, identifying methods to cope with this phenomenon is as important as measuring its burden. Therefore, health facilities should consider proper educational burnout management programs in order to achieve promising outcomes regarding nurses’ mental well-being. The first limitation of this study was the self- reported survey, and as a result, there may be a reporting bias. Second, a causal inference cannot be made because of the cross-sectional nature of this study. Third, response rate was reasonably decreased since the authors were limited in approaching the nurses due to the COVID-19 aura. Finally, the sample did not contain an equal number of males and females, with the latter dominating, which may limit drawing conclusions regarding gender as a contributing factor.

## Conclusion

The study showed that most nurses either fit the criteria of burnout or are at a high risk of it. Adequate staffing and psychological consultations are recommended in high workload units to decrease burnout among nurses.

## Data Availability

The datasets generated and/or analyzed during the current study are not publicly available due to respondents’ confidentiality but are available from the corresponding author on reasonable request.
